# Review of the current ongoing clinical trials exploring the possible anti-anxiety effects of cannabidiol

**DOI:** 10.1186/s42238-024-00250-y

**Published:** 2024-10-12

**Authors:** Rhenu Bhuller, Walter K. Schlage, Julia Hoeng

**Affiliations:** 1 Vectura Fertin Pharma Switzerland SA, Avenue de Rhodanie 50, Lausanne, 1007 Switzerland; 2 Biology Consultant, Max-Baermann-Strasse 21, Bergisch Gladbach, 51429 Germany

**Keywords:** Anxiety disorders, Cannabidiol, Clinical trials, Narrative review

## Abstract

**Background:**

Anxiety disorders (ADs) are a complex group of mental disorders and majorly contribute to the global health-related burden. Symptoms and clinical management differ widely depending on the specific diagnosis. There is a need for new, more effective pharmacological treatments for these patients as many patients do not respond to treatment and treatment is not available for several types of AD. The increased interest in the potential effects of cannabidiol (CBD) on symptoms of AD has led to several preclinical and clinical studies that suggest that CBD may be effective in some patients with AD. However, it remains unclear whether and how CBD can be used in the clinical management of ADs due to a lack of sufficiently robust clinical evidence.

**Comparative evaluation:**

This narrative review provides a critical analysis of the current state of the art for ADs and summarizes six recently completed and 22 currently ongoing clinical trials investigating the effects of CBD on ADs or anxiety. The aim was to examine whether the ongoing trials are likely to provide the necessary solid evidence, or whether new studies with more robust design parameters can help to overcome the prevailing lack of solid clinical data for this CBD indication. Most of the trials reviewed are considered exploratory and do not focus on specific types of clinical anxiety or ADs as the primary condition studied. Participant numbers, CBD dose, treatment duration, and CBD formulation vary widely among the studies, and all but two are single-center studies.

**Conclusion:**

For an effective clinical management of ADs using CBD, there is a need for sufficiently powered and appropriately designed clinical trials (RCT, multicenter, defined doses and exposure monitoring, robust primary outcomes) investigating the effect of CBD in specific ADs, such as social anxiety disorder and panic disorder, or in post-traumatic stress disorder.

## Introduction

Anxiety disorders (ADs) represent a leading cause of disability-adjusted life years (DALYs) and—together with major depressive disorder (MDD)—one of the 25 leading causes of the current global health-related burden. In 2013, the global prevalence of ADs was estimated to be 7.3% (Baxter et al. 2013), and large population-based surveys report that up to 33.7% of the population in the United States of America (US) and 14.5% of the population in Europe experience an AD at some point in life (Alonso & Lépine 2007; Kessler et al. 2012). Between 2010 and 2021, global age-standardised DALY rates increased most substantially for anxiety disorders (16·7% [14·0–19·8]) and depressive disorders (16·4% [11·9–21·3]). As of 2019, cumulatively, 301 million people worldwide were living with ADs (World Health Organization (WHO), 2022b).

The World Health Organization (WHO) reported that, during the first year of the COVID-19 pandemic, there was a 25% increase in the global prevalence of AD and MDD (World Health Organization (WHO), 2022a)). One possible explanation for the increased prevalence and burden of AD is the stress caused by social isolation during the pandemic.

According to the Diagnostic and Statistical Manual of Mental Disorders, 5th edition, (DSM-5) (American Psychiatric Association (APA), 2013). ADs are listed as some of the most difficult mental disorders to correctly diagnose, given the normal and adaptative nature of fear, avoidance, and anxiety in human behavior. Furthermore, ADs have an earlier age of onset than mood disorders and are more prevalent in female than male patients (Bandelow & Michaelis 2015). An apparent under-representation of females in the preclinical research and a lack of female AD patients in clinical trials has already previously been observed, and more efforts to close the knowledge gap on gender differences in the effectiveness of cannabidiol (CBD) in AD treatment have been postulated (Wright et al. 2020).

A differential diagnosis of AD can be made based on the guidelines presented in the International Classification of Diseases (ICD-11) (World Health Organization (WHO), 2021) or the DSM-5 (American Psychiatric Association (APA), 2013). Detailed information regarding the classification of ADs and stress-related disorders according to these two sources is shown in Table 1.

**Table 1:** Current classification of anxiety disorders

DSM-5^a^	ICD-11^b^
**Anxiety disorders** • Separation anxiety disorder • Selective mutism • Specific phobia • Social anxiety disorder (social phobia) • Panic disorder • Panic attack (specifier) • Agoraphobia • Generalized anxiety disorder • Substance/medication-induced anxiety disorder • Anxiety disorder due to another medical condition • Other specified anxiety disorder • Unspecified anxiety disorder **Obsessive–compulsive and related disorders** • Obsessive–compulsive disorder • Body dysmorphic disorder • Hoarding disorder • Trichotillomania (hair-pulling disorder) • Excoriation (skin-picking) disorder • Substance/medication-induced obsessive–compulsive and related disorder • Obsessive–compulsive and related disorder due to another medical condition • Other specified obsessive–compulsive and related disorder • Unspecified obsessive–compulsive and related disorder **Trauma- and stress-related disorders** • Reactive attachment disorder • Disinhibited social engagement disorder • Post-traumatic stress disorder • Acute stress disorder • Adjustment disorders • Other specified trauma- and stressor-related disorder • Unspecified trauma- and stressor-related disorder	**Anxiety and fear-related disorders** • Generalized anxiety disorder • Panic disorder • Agoraphobia • Specific phobia • Social anxiety disorder • Separation anxiety disorder • Selective mutism **Obsessive–compulsive disorders** • Body dysmorphic disorder • Olfactory reference disorder • Hypochondriasis • Hoarding disorder • Body-focused repetitive behavior disorders **Disorders specifically associated with stress** • Post-traumatic stress disorder • Complex post-traumatic stress disorder • Prolonged grief disorder • Adjustment disorder • Reactive attachment disorder • Disinhibited social engagement disorder • Other specified disorders specifically associated with stress • Disorders specifically associated with stress, unspecified

^a^Includes obsessive–compulsive and trauma related disorders

^b^Includes fear-related disorders, obsessive disorders, and traumatic disorders

*DSM-5* Diagnostic and Statistical Manual of Mental Disorders, Fifth Edition (American Psychiatric Association (APA), 2013); *ICD-11* International Classification of Diseases, Eleventh Revision (World Health Organization (WHO), 2021)

The pharmacological treatment of ADs may be personalized to some extent, based on the specific diagnosis along with the response and tolerance of each patient. Currently available treatments also have limitations: with chronic use, some are associated with dependence and withdrawal syndrome, and a significant proportion of patients experience incomplete or no response after using these drugs (Cipriani et al. 2018; Roy-Byrne 2015; Sarangi et al. 2021).

In light of such limitations, the scientific and medical community are continually seeking new and more effective treatments for AD. CBD, the major non-psychotomimetic compound of the *Cannabis sativa* plant, has demonstrated anti-anxiety properties in healthy volunteers submitted to experimental anxiety models and in patients diagnosed with ADs (Crippa et al. 2018; Sarangi et al. 2021). In a previous review, the authors concluded that, in spite of promising preliminary evidence of CBD’s anxiolytic effects in social anxiety disease (SAD), “future research is warranted to determine the efficacy of CBD in other anxiety disorders, establish appropriate doses, and determine its long-term efficacy.” (Wright et al. 2020). Likewise, a recent review of 58 published clinical trials on CBD as a treatment for non-convulsive diseases (including eight identified AD trials) noticed that “… the effectiveness of CBD is unclear and additional research with larger sample sizes are paramount to elucidate these relationships.” (Tang et al. 2022).

In this review, we provide a brief overview of the current state of treatment options for ADs and critically discuss the strength of evidence available from recently completed and ongoing or planned clinical trials investigating the clinical effectiveness of CBD for the treatment of ADs.

### Treatments for AD: current and future directions

Anxiety is a normal and adaptative emotional state triggered by a threat that leads to the expression of defensive behaviors (approach to danger, risk assessment, and passive avoidance) (Gray & McNaughton 2003). However, when this adaptative behavior reaches high levels of activation even in the absence of a potential and real threat, anxiety changes from a normal emotion to a psychiatric condition, needing specific diagnosis, psychological support, and pharmacological treatment (Gray & McNaughton 2003).

It is important to note that few patients with ADs seek treatment. In a European study, among individuals with ADs who sought treatment (approximately 21%), only around 20% received psychological treatment, 31% received pharmacological treatment, and 27% received both types of intervention; these proportions may be much lower for patients with specific phobias (Alonso & Lépine 2007).

In the early part of the twentieth century, when all ADs were classified as neurosis, barbiturates were introduced as the first line of treatment for ADs. Although they produced potent effects in reducing anxiety, they posed a risk to patients’ lives because of their narrow pharmacological window and toxic effects. In the 1960s, benzodiazepines (BZDs) were discovered, replacing most prescriptions of barbiturates for AD (Sternbach 1979). At the same time, another class of anti-anxiety drugs was discovered: antidepressants. Together, BZDs and antidepressants became the first-line treatment for AD (Bandelow et al. 2017). As detailed in Table 1, there are different possible diagnoses of AD, so different treatments have been prescribed according to each patient’s symptoms and tolerance (for a review, see (Bandelow, Allgulander, et al., 2022; Bandelow et al. 2017)). However, BZDs are associated with adverse effects, such as fatigue, dizziness, increased reaction time, and impaired driving, and when used chronically, patients can develop dependence and withdrawal symptoms (Bandelow et al. 2017), which has led to the downgrading of BZDs to secondary treatment in the most recent World Federation of Societies of Biological Psychiatry guidelines (Bandelow, Allgulander, et al., 2022).

Following diagnosis, first-line treatment can follow one of the two main pathways: psychotherapy and pharmacotherapy. Psychotherapy includes psychological interventions such as cognitive behavioral therapy, psychodynamic therapy, and acceptance and commitment therapy. Pharmacotherapy includes medications, of which nowadays antidepressants, typically selective serotonin reuptake inhibitors (SSRIs) and selective serotonin-noradrenalin reuptake inhibitors (SNRIs), are used as first-line interventions. In the US, either of the modalities of treatment, i.e., pharmacotherapy or psychotherapy, can be started in patients with ADs, and combination therapy is reserved as a second-line intervention in case of non-response, progression of disease, or relapse (Baldwin et al. 2018). In the UK, as per National Institute for Health and Care Excellence (NICE) guidelines, individual cognitive behavioral therapy is the initial choice of treatment after first diagnosis. The second line of therapy is pharmacological intervention, with SSRIs and SNRIs being the preferred options, followed by combination therapy in cases of non-response or progression (National Institute for Health and Care Excellence (NICE), 2020). Most European countries, including Spain, Italy, and Germany, follow either US or NICE guidelines (Bandelow, Allgulander, et al., 2022; Bandelow, Werner, et al. 2022; Guideline Working Group for the Treatment of Patients with Anxiety Disorders in Primary Care 2008; Psicoterapia Scientifica (n.d.)).

As first-line treatment, patients with generalized anxiety disorder (GAD) may be prescribed SSRI or SNRI antidepressants, agomelatine (slow-release melatonin) has also been considered (Bandelow, Werner, et al. 2022). For panic disorder, agoraphobia, separation anxiety, obsessive–compulsive disorder (OCD), and some stress-related disorders, antidepressants are also the first choice (Bandelow, Allgulander, et al., 2022; Bandelow et al. 2012). They are effective particularly in patients with panic disorder or agoraphobia, but provide incomplete or no clinical effects for patients with other forms of AD. Unfortunately, antidepressants are associated with a delayed onset of clinical effectiveness, often not providing benefit to patients until they have been on treatment for 6–12 weeks. They are also associated with adverse effects such as jitteriness, nervousness, and insomnia (Bandelow et al. 2017). The clinical failure rate is approximately 25% (Bandelow et al. 2017; Cipriani et al. 2018).

As emphasized by the WHO, there is a need to improve support for mental health including the discovery of new and more effective treatments for ADs (World Health Organization (WHO), 2022b).

There s a rapidly growing interest in the medical literature in the putative effects of cannabinoids, specifically CBD, on AD. Several preclinical and clinical studies, mainly consisting of small trials and case reports, suggest that CBD is effective as an anti-anxiety drug (Crippa et al. 2018). However, it remains unclear whether CBD and other cannabinoids could be used in the clinical management of ADs, especially given the complex nature and differing characteristics of ADs and the fact that multiple disorders with heterogeneous etiologies are classified under the umbrella of ADs.

### Cannabidiol and anxiety: scientific evidence

CBD is the major non-psychotomimetic phytocannabinoid with anti-anxiety properties (Crippa et al. 2018). Early reports suggested that, in human volunteers who received a high dose of the psychotomimetic cannabinoid Δ^9^-tetrahydrocannabinol (THC), CBD blocked the anxiogenic responses of THC (Zuardi et al. 1982). This observation raised the hypothesis that CBD could produce anxiolytic effects when administered to patients.

In 1993, a study using healthy volunteers submitted to experimental anxiety was published by (Zuardi et al. 1993) and reported a positive effect of CBD compared with placebo, an effect that was similar to the BZD diazepam. The putative anxiolytic properties of CBD were later characterized in patients with social anxiety with positive results (Bergamaschi et al. 2011; Linares et al. 2019).

Preclinical models using rodents have demonstrated that CBD produced anxiolytic-like effects with bell-shaped dose–response curves; i.e., whereas intermediate doses (e.g., 5–10 mg/kg in rats) produced positive effects, the anti-anxiety effects disappeared at a low dose (2.5 mg/kg in rats) and high dose (20 mg/kg in rats) (Campos et al. 2012). The preclinically observed bell-shaped dose profile of the anti-psychotic effect of CBD was clinically confirmed in social phobia patients and in healthy volunteers in a model of social anxiety, the public speaking test (Bergamaschi et al. 2011; Linares et al. 2019; Zuardi et al. 2017). However, the bell-shaped dose profile of acute CBD treatment was not observed in five trials on CBD-treated anxiety patients, as recently reviewed (Narayan et al. 2022), who reported that similar anxiolytic effects were achieved by low as well as high doses (18–800 mg/day) in chronic treatment settings. This heterogeneous dose–response behavior, and the low and variable bioavailability of CBD is a critical determinant for dose selection, which must be taken into consideration when planning clinical trials. Robust experimental designs may need to consider the individual effective dose (e.g., plasma level) in order to optimize the personal dose of CBD. Therefore, although several studies indicate that CBD can produce anti-anxiety effects, few clinical trials to date have assessed the role of CBD in large numbers of patients with different specific AD diagnoses, and positive outcomes from these trials have not yet been reported. This lack of solid clinical evidence as also observed in previous reviews (Tang et al. 2022; Wright et al. 2020) has triggered our reviewing of the recently completed and ongoing clinical trials and a critical examination of parameters that may influence the outcomes of these studies, as well as help designing future successful studies. Our aim was to obtain information whether the still ongoing trials are likely to provide such more solid evidence, or whether new studies with more robust design parameters can offer an increased potential to overcome the prevailing lack of solid clinical data for this CBD indication.

### Recently completed and currently ongoing clinical trials investigating CBD for the treatment of ADs—overview

A combined search strategy was conducted for this narrative review to search clinical trials posted on ClinicalTrials.gov, as well as a database search, using the following keywords: ‘cannabidiol’ and ‘anxiety’ at ClinicalTrials.gov., or ‘cannabidiol’ and ‘anxiety’ and ‘clinical trials’. Although post-traumatic stress disorder (PTSD) is included under the separate category of trauma- and stressor-related disorders in the DSM-5, it has been subsumed under the category of ADs for the purposes of this review, given the close relationships between the two groups of disorders.

Thirty-five matching records were found (see Table 2). Of those, five studies were withdrawn or terminated, with reasons such as the COVID-19 pandemic, a change in the drug pipeline, or a decision to terminate the study prior to participant recruitment; two studies are classified with unknown status; six studies were completed but for three of them, results were not yet posted at the time of writing (April 2024); three studies were completed and had results posted. The remaining, currently ongoing studies were not yet recruiting (six), in the recruiting phase (15), or active but not recruiting (two).

**Table 2:** Clinical trials on the effect of CBD treatment on AD

CT registration No	Study title	Status (Apr.2024)	Study completion (actual or estimated)	Study type/design	Number of participants (actual or estimated)	AD type/symptoms	CBD Treatment	Treatment duration	Reference link
**Completed trials**									
NCT05003882	Radicle ACES: A study of commercially available CBD used in the real-world setting	completed, no results posted	31-Dec-21	13-arm, real-world, open-label study	3000	anxiety, pain, sleep disturbance	12 different commercial CBD extracts	4 weeks	https://classic.clinicaltrials.gov/ct2/show/NCT05003882
NCT05108220	Evaluation of effects of CBD products among US women	completed, no results posted	30-Dec-20	retrospective, observational study	nine groups, 150 female participants each (total, *N* = 1350)	GAD in women	8 different CBD products, 15 to 30 mg/day	8 weeks	https://classic.clinicaltrials.gov/ct2/show/NCT05108220
NCT05253417	The CANabidiol Use for RElief of Short-Term insomnia (CANREST)	completed, no results posted	07-Jul-23	double-blind, randomized, parallel-group, placebo-controlled study	208	Insomnia due to anxiety and fear, and other forms of insomnia	oral capsule, 50 or 100 mg per day	8 weeks	https://classic.clinicaltrials.gov/ct2/show/NCT05253417
NCT02818777	A study of the tolerability and efficacy of cannabidiol on tremor in Parkinson’s disease	completed, results posted	Nov-17	open-label, single-group assignment	13	Focus on Parkinson's disease symptoms, an anxiety questionnaire was included (no CBD effects on AD reported)	Epidiolex^®^, started at 5 mg/kg/day, increased up to 20 mg/kg/day	5 weeks	https://classic.clinicaltrials.gov/ct2/show/NCT02818777
NCT03582137	A study of tolerability and efficacy of cannabidiol on motor symptoms in Parkinson´s disease	completed, results posted	04-Jan-22	randomized, double-blind, placebo-controlled (parallel group) design, Phase 2	74	Focus on Parkinson's disease symptoms, an anxiety questionnaire was included (no CBD effects on AD reported)	CBD extract (oral) or placebo, titrated up to 2.5 mg/kg/day	2–3 weeks	https://classic.clinicaltrials.gov/ct2/show/NCT03582137
NCT05023759	Anxiety symptoms in relation to use of hemp-derived, full-spectrum CBD	completed—no results posted	31-Oct-20	open-label, observational prospective study	30	GAD	oral capsules, full-spectrum CBD extract, 25 mg/day	8 weeks	https://classic.clinicaltrials.gov/ct2/show/NCT05023759
**Currently ongoing trials**									
NCT04482244	RCT of CBD for anxiety in advanced breast cancer	active, not recruiting	18-Dec-24	randomized, double-blind, placebo-controlled Phase 2 trial of a single dose of CBD	50	Acute anticipatory anxiety in Advanced Breast Cancer patients	Epidiolex^®^, per protocol dosage	single acute dose	https://classic.clinicaltrials.gov/ct2/show/NCT004482244
NCT05600114	Cannabidiol (CBD) for the Treatment of Social Anxiety Disorder	active, not recruiting	Dec-23	multicenter, double-blind, parallel group, placebo-controlled, randomized phase 2 trial	225	SAD	oral solution, 300 or 600 mg/day	10 weeks	https://clinicaltrials.gov/study/NCT05600114
NCT05823753	Cannabidiol to Reduce Anxiety Reactivity	recruiting	Apr-25	Randomize, placebo-controlled 3-arm, sub-acute (4-day) steady state dosing phase 2 clinical trial	60	SAD	Epidiolex^®^, 300 mg/day or 900 mg/day	4 days	https://clinicaltrials.gov/study/NCT05823753
NCT05753007	A Clinical Trial of a Hemp-Derived, High Cannabidiol Product for Anxiety in Glioblastoma Patients	recruiting	Sep-25	double-blind, placebo-controlled, 8-week randomized phase 2 trial	36	Anxiety, pain, QOL in glioblastoma patients	Full-spectrum, hemp-derived, "ultra-high CBD" solution, dose not indicated	8 weeks	https://clinicaltrials.gov/study/NCT05753007
NCT06364254	Effects of CBD on 2 Mile Run Time Trial Performance	recruiting	30-May-24	cross-over, placebo-controlled pilot study	30	pre-race anxiety and physical symptoms in running women	Oral capsule, 300 mg, 2 h prior to 2 mile run	single acute dose	https://clinicaltrials.gov/study/NCT06364254
NCT03944447	Outcomes Mandate National Integration With Cannabis as Medicine (OMNI-Can)	recruiting	31-Dec-25	multistate, multicenter clinical phase 2 study	200,000	variety of 33 chronic debilitating conditions including anxiety	medical cannabis (RYAH-Medtech Inhaler) dosing will be recommended depending on indication	5 years or longer	https://clinicaltrials.gov/study/NCT03944447
NCT05457465	Assessing the Impact of Cannabidiol for Anxiety and Depression in Bipolar Disorder	recruiting	Jun-25	open-label clinical phase 2 trial, pilot study	25	anxiety, depression in bipolar disorder patients	sublingual, hemp-derived, high-CBD product, daily dose not indicated	4 weeks	https://clinicaltrials.gov/study/NCT05457465
NCT05571592	Cannabidiol as a Treatment for Social Anxiety Disorder (R61)	recruiting	01-Aug-24	double-blind randomized controlled phase 2 trial	60	SAD	softgel capsules with self-emulsifying nanoformulation: 400 mg/day or 800 mg/day	3 weeks	https://clinicaltrials.gov/study/NCT05571592
NCT04978428	Epidiolex in obsessive compulsive disorder and related disorders	recruiting	01-Sep-24	open-label, single-group intervention trial (proof-of-concept)	15	Obsessive compulsive disorder and related disorders. Anxiety not explicitly included	Epidiolex^®^ (2.5 mg/kg b.i.d for one week followed by 5 mg/kg b.i.d.) for two total weeks	3 weeks	https://classic.clinicaltrials.gov/ct2/show/NCT04978428
NCT03948074	Cannabis For Cancer-Related Symptoms (CAFCARS)	recruiting	01-Mar-24	randomized, double-blind, placebo-controlled phase 2 study	150	Anxiety, nausea, pain, sleep disturbance in cancer patients	3 different Cannabis oil extracts: High THC/Low CBD, Low THC/High CBD, and 1:1 THC/CBD, titrated for up to 18 drops/day (dose not indicated)	1 to 4 cycles of 16 days each	https://clinicaltrials.gov/study/NCT03948074
NCT03549819	Cannabidiol for the Treatment of Anxiety Disorders: An 8-Week Pilot Study	recruiting	Feb-24	randomized, triple-blind, phase 3 trial that uses a parallel assignment model, implying a two-arm trial (CBD or placebo)	50	GAD, SAD, panic disorder, and agoraphobia	CBD oral capsules, 200 to 800 mg/day	8 weeks	https://classic.clinicaltrials.gov/ct2/show/NCT03549819
NCT02548559	Sublingual CBD for anxiety disorders	recruiting	Aug-25	randomized, open-label to double-blind, placebo-controlled phase 1/2 study	approx. 100	various AD, not specified	CBD full spectrum extract or pure CBD, sublingual; 30 mg/day	4 weeks	https://classic.clinicaltrials.gov/ct2/show/NCT02548559
NCT04075435	Cannabidiol solution for the treatment of behavioral symptoms in older adults with Alzheimer’s disorder	recruiting	15-Sep-24	single-group assignment open-label phase 1 study	12	anxiety in Alzheimer Disease patients	high CBD/low THC sublingual solution, variable dosing	8 weeks	https://classic.clinicaltrials.gov/ct2/show/NCT04075435
NCT05324449	Epidiolex^®^ for anxiety in pediatric epilepsy	recruiting	04-Apr-26	open label, adjunctive, proof of concept, prospective phase 4 clinical trial	20	anxiety secondary to refractory pediatric epilepsy	Epidiolex^®^, flexible dose titration; adjunct to anticonvulsive treatment	16 weeks	https://classic.clinicaltrials.gov/ct2/show/NCT05324449
NCT02283281	Anesthetic premedication with cannabis extracts (Cannapremed)	recruiting	Dec-24	randomized, placebo-controlled (parallel assignment with three arms) phase 2/3 trial	200	Perioperative Anxiety, postoperative pain, nausea & vomiting	Cannabis oil, intravenous, single dose (21.6 mg THC + 20 mg CBD) or (10.8 mg THC + 10 mg CBD)	single acute dose	https://classic.clinicaltrials.gov/ct2/show/NCT02283281
NCT04878627	Role of CBD in regulating meal-time anxiety in anorexia nervosa	recruiting	Oct-24	double-blind, placebo-controlled (parallel assignment) phase 1 study	40	Anxiety in anorexia nervosa patients	Epidiolex^®^, dose titration from2.5 mg/kg to 7.5 mg/kg	3 weeks	https://classic.clinicaltrials.gov/ct2/show/NCT04878627
NCT05283382	Cannabidiol effects on learning and anxiety	not yet recruiting	Feb-25	randomized, double-blind, placebo-controlled trial with two-by-two factorial assignment	160	healthy volunteers who display elevated social anxiety	one 600 mg CBD Isolate Gel Capsule	single acute dose	https://classic.clinicaltrials.gov/ct2/show/NCT05283382
NCT06290063	Cannabidiol and Older Adult Cannabis Users (QUARTz)	not yet recruiting	31-Aug-28	3-arm randomized, placebo- controlled phase 2 trial	385	anxiety in older individuals who seek to use cannabis	oral capsules, full-spectrum (200 mg CBD, 4 mg THC) or broad spectrum (200 mg CBD, 0 mg THC) CBD capsules, 200 mg/day	8 weeks	https://clinicaltrials.gov/study/NCT06290063
NCT06266611	Cannabis for Palliative Care in Cancer (ARCTiC)	not yet recruiting	31-Jul-28	3-arm, placebo-controlled randomized phase 2 trial	185	Anxiety and other symptoms in palliative cancer patients	oral capsules, full-spectrum (200 mg CBD, 4 mg THC) or broad spectrum (200 mg CBD, 0 mg THC) CBD capsules, 200 mg/day	8 weeks	https://clinicaltrials.gov/study/NCT06266611
NCT06123702	Cannabidiol Effects on Fear Extinction in Social Phobia	not yet recruiting	30-Sep-24	double-blind, randomized controlled phase 1 trial	20	SAD	oral capsules, 600 mg/day	single acute dose	https://clinicaltrials.gov/study/NCT06123702
NCT05649059	Investigating the Effects of Cannabidiol on Social Anxiety Disorder	not yet recruiting	May-25	randomized, double-blind, placebo-controlled, parallel-group phase 4 trial	50	SAD	Epidiolex^®^, 300 mg	single acute dose	https://clinicaltrials.gov/study/NCT05649059
NCT06261502	Effect of CANnabidiol on Anxiety and GABAergic Function in Individuals With Fragile-X Syndrome (CANAX)	not yet recruiting	Aug-27	Randomized, double-blind, placebo-controlled, single center, cross-over phase 2 trial	40	Anxiety, GABAergic function in Fragile X patients	Oral solution (Epidiolex^®^?), escalating from 5 mg/kg/day to 10 mg/kg/day	2 × 12 weeks, 8-week washout	https://clinicaltrials.gov/study/NCT06261502
**Abandoned trials**									
NCT05429788	Safety, tolerability, and efficacy of RLS103 in subjects with acute anxiety with social anxiety disorder	Withdrawn	08-Nov-22	randomized, double-blind, placebo-controlled (parallel assignment) phase 1b/2a trial	30	SAD	CBD inhalation powder (RLS103), 3 mg or 6 mg CBD per administration	single acute dose	https://classic.clinicaltrials.gov/ct2/show/NCT05429788
NCT04267679	Cannabidiol for anxiety	withdrawn	01-Dec-20	single-group assignment, open-label phase 2 trial (pilot)	not indicated	GAD	full-spectrum CBD soft gel capsules, up to 100 mg/day	12 weeks	https://classic.clinicaltrials.gov/ct2/show/NCT04267679
NCT04286594	A clinical trial of hemp-derived CBD product for anxiety	terminated by sponsor	24-Aug-23	Phase 2 randomized open-label to double-blind study	12	Anxiety (not specified)	sublingual CBD solution (hemp extract), 30 mg/day	6 weeks	https://classic.clinicaltrials.gov/ct2/show/NCT04286594
NCT04726475	Impact of CBD-rich oil on aversive memory reconsolidation	Unknown status	Jan-24	three-arm, proof-of-concept double-blind, placebo-controlled trial phase 1/2 trial	96	PTSD (anxiety & fear)	CBD-rich hemp extract oil, acute dose 300 mg	two single doses	https://classic.clinicaltrials.gov/ct2/show/NCT04726475
NCT04729244	The study of hemp oil CBD for evaluation of efficacy and safety in treatment of pain, anxiety, and insomnia management	Unknown status	01-Feb-22	non-randomized, open-label pilot study	30	Chronic pain, anxiety and insomnia	CBD tincture or cream, 50 mg/day	4 weeks	https://classic.clinicaltrials.gov/ct2/show/NCT04729244
NCT04569760	Cannabidiol for the treatment of anxiety disorders: an 8-week pilot study	withdrawn	Jun-23	randomized, double-blind, placebo-controlled parallel design, phase 3 (pilot study)	50	GAD, SAD, panic disorder, and agoraphobia	50:2 mg(CBD:THC)/ml—Cannabinoid Oil Oral Preparation; titrated (200 mg- 800 mg total CBD dose)	8 weeks	https://classic.clinicaltrials.gov/ct2/show/NCT02283281
NCT04086342	CHI-902 for treatment of social anxiety disorder	withdrawn	26-Jan-21	randomized double-blind, placebo-controlled phase 2 trial	160	SAD	CBD extract in MCT oil; titration from 150 to 300 mg/day	4 weeks	https://classic.clinicaltrials.gov/ct2/show/NCT04086342

Overview and brief synthesis of the selected trials from ClinicalTrials.gov. For a detailed description, see the text sections on Recently completed and currently ongoing clinical trials investigating CBD for the treatment of ADs

In general, the trials were quite heterogeneous in study design, duration, inclusion criteria and number of participants, CBD formulation and dosing. Information regarding the origin of CBD formulations and daily doses of CBD used tended to be incomplete in several studies. Most studies used a single dose level and formulation of CBD; eight studies used Epidiolex^®^. The doses described or used in 16 of these clinical trials were either undefined or quite low compared with doses of CBD that have been reported to alleviate anxiety symptoms (e.g., 300–600 mg/day pure CBD in corn oil (Crippa et al. 2018)(Crippa et al. 2018)), and it was unclear for some of the trials whether the investigators planned to use fixed doses of pure CBD or CBD-rich cannabinoid products (i.e., full spectrum and extracts). In several studies, there was no clear definition (AD-specific subjective scores in questionnaires, inclusion criteria) of a single or prevalent AD symptom to be targeted. Outcome measurements were often limited to one or several standardized questionnaires (see Table 3 for definitions of the most prevalent scoring instruments, additional scores are summarized in (Narayan et al. 2022)). Furthermore, 14 of the trials have a low number of planned participants (< 50 total participants). Only eleven of these ongoing/planned trials employ a randomized controlled trial (RCT) design, which is the gold standard for clinical trials evaluating pharmacological treatments. For most studies, either the included patients were required to be on stable pharmacological treatment, or no information regarding concomitant medications was provided. In cases where the information is available, it appears that CBD was only being tested as an add-on treatment. For the majority of the clinical trials identified, there was no clear representation of women and young adults, which are the populations most likely to be diagnosed with ADs. Only two of the trials identified are true multicenter trials (one RCT and one open-label trial) and very few plan to analyze plasma concentrations of CBD and its metabolites or other cannabinoids and their metabolites. Oral formulations (oil or capsules) are used in approximately 90% of the trials.

**Table 3:**
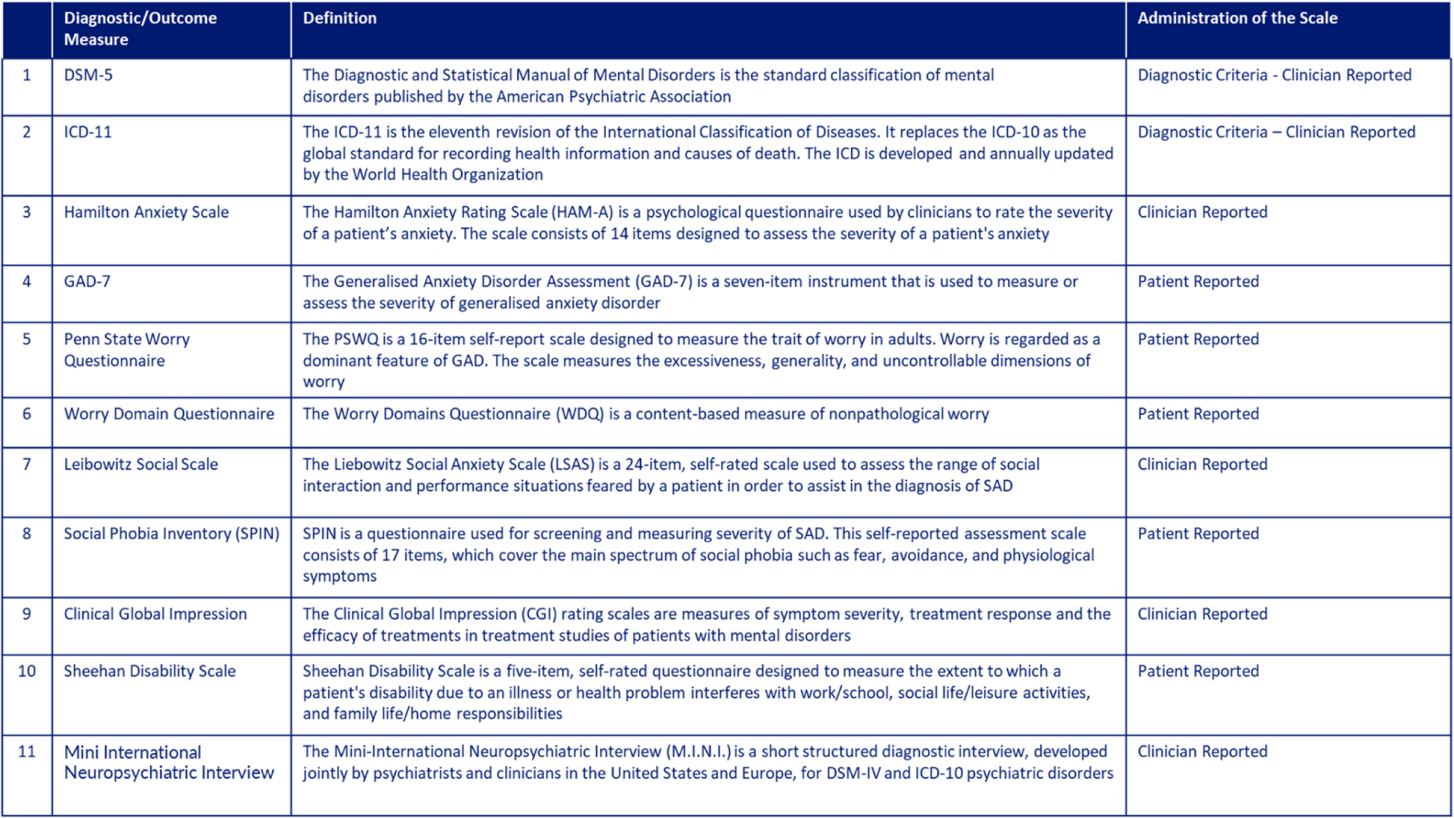
Diagnostic & Outcome Measures Definitions^a^

^a^This list is not exhaustive and is based on the most common outcome measures

Table 3 was kindly provided by Syneos Health in an analysis of data from ClinicalTrials.gov, Biomedtracker, and PubMed, and particularly from the following references: (European Medicines Agency (EMA), 2006; Lecrubier et al., 2000; Stein & Stein, 2008)

The following sections will review in greater detail the recently completed and currently ongoing clinical trials registered at ClinicalTrials.gov, outlining the points of interest and potential limitations of each study in the context of CBD use for the treatment of patients with ADs in the clinical setting. Abandoned studies have also been included because some of them had a robust design or aimed at testing novel routes of administration (transdermal, inhaled). For a brief synthesis and overview, see Table 2.

### Recently completed clinical trials investigating CBD for the treatment of ADs: study details

#### NCT05003882: Radicle ACES: A study of commercially available CBD used in the real-world setting; complete, no results posted

This 13-arm, real-world, open-label study aimed to determine the size of the effect of commercially available CBD products on a primary outcome of overall well-being, and secondary assessments focusing on measures of pain, sleep quality, and self-reported anxiety (as assessed by GAD-7, Patient Reported Outcomes Measurement Information Systems Anxiety 4A) over a period of 28 days. A total of 3000 participants were included (a probability sample was used) and multiple commercial CBD products were tested.

While the number of participants included in the study was relatively large, the focus of the study was the effects of CBD on subjective anxiety in the general population. Thus, this study has reduced relevance in the context of CBD use for the clinical treatment of ADs.

#### NCT05108220: Evaluation of effects of CBD products among US women; completed, no results posted

This retrospective, observational study includes nine groups comprising 150 participants each (total, *N* = 1350). The trial used the GAD-7 scale to analyze the prevalence of general anxiety in women aged 24–44 years, which is the population most susceptible to GAD. The investigators explored different compositions of CBD oils, which included different concentrations of CBD with or without a low percentage of THC (< 0.3%). A 60-day follow-up is expected to be adequate for this type of retrospective study.

The intervention is based on a dietary supplement containing CBD, making it potentially difficult to predict the actual daily dose of CBD or THC received by participants. The study did not exclude participants with other psychiatric or neurological conditions, which may complicate the interpretation of the results. This study only evaluated a single primary outcome, with no secondary outcomes listed.

#### NCT05253417: The CANabidiol Use for RElief of Short-Term insomnia (CANREST); completed, no results posted

This double-blind, placebo-controlled (parallel assignment), randomized phase 2 study is planned to have three arms and include 198 participants with insomnia either as their main condition or associated with a mental disorder, including ADs. Two different doses of pure CBD (50 mg/day and 100 mg/day) delivered in a special type of capsule (BodECS BioAbsorb™) will be evaluated. Although little information could be found regarding the capsule, we surmise from the name that it is likely planned to be used with the objective of enhancing the absorption of CBD, which is an important consideration. The primary outcome of evaluating the effect of CBD on insomnia using the Insomnia Severity Index is quite appropriate, as insomnia and other sleep disturbances are among the most frequently reported symptoms in patients with ADs (Staner 2003). The secondary outcomes of change in wake after sleep onset, and stress and anxiety outcomes (assessed using the Depression Anxiety Stress Scale-21) are also highly relevant. The inclusion and exclusion criteria are suitable for the study objectives.

The specific types of AD that are considered associated with insomnia in this study are not detailed and there is no information on the origin of the CBD, though it is noted that it is not approved by the US Food and Drug Administration.

#### NCT02818777: A study of the tolerability and efficacy of cannabidiol on tremor in Parkinson’s disease; complete, results posted

Participants were being treated with levodopa for Parkinson’s disease during the trial. Plasma levels of metabolites of both CBD and levodopa were measured. Pharmaceutical grade CBD was used in the study (Epidiolex^®^).

This study design was an open-label, single-group assignment, which has some inherent limitations. Additionally, the purpose of the study was to evaluate the effects of CBD on tremor in Parkinson´s disease patients; therefore, in the context of ADs, this study has less value as evaluation of AD was not a primary outcome (although anxiety symptoms were assessed using the Anxiety Short Form, and emotional effects were assessed using the Emotional and Behavioral Dyscontrol Short Form). The study included 13 participants and not all of them reported anxiety as a symptom or as a secondary condition (i.e., AD). A dose escalation protocol was used (initial dose, 5 mg/kg/day for 5 days; dose escalation, 20 mg/kg/day).

The results of this study were recently published (Leehey et al. 2020). A small but positive effect of the higher dose of CBD (20 mg/kg/day) was observed on tremors measured by the Movement Disorder Society-Unified Parkinson’s Disease Rating Scale. There were significant improvements in nighttime sleep and emotional/behavioral dyscontrol scores. There were no reported effects on specific anxiety symptoms.

#### NCT03582137: A study of tolerability and efficacy of cannabidiol on motor symptoms in Parkinson´s disease; complete, results posted

The study had a randomized, double-blind, placebo-controlled (parallel group) design and enrolled 74 participants who received either CBD extract as an oral solution or placebo.

As this study was focused on evaluating the effects of CBD on motor symptoms in patients with Parkinson’s disease, the study did not directly assess patients with ADs. However, one of the secondary outcomes was to assess change in anxiety from baseline up to 3 weeks using the Anxiety Short Form response. This trial is conducted by the same group as NCT02818777 and appears to be a follow-up to that study. In both trials, the posted results indicate no effect of CBD on anxiety. Recently, some results of the study have been published (Domen et al. 2023; Liu et al. 2024), but anxiety results have not been mentioned.

#### NCT05023759: Anxiety symptoms in relation to use of hemp-derived, full-spectrum CBD; completed, no results posted

This trial focused on a single AD, GAD, and used the GAD-7 to evaluate change from baseline at week 8. Medical care providers provided weekly clinical assessments throughout the study. The inclusion and exclusion criteria were appropriate for the study.

As an observational prospective study, this study did not include a placebo group. Additionally, the number of participants included was quite small (*N* = 30), which may have impacted the statistical power of the study. Finally, the CBD was not pharmaceutical grade and was given as a dietary supplement at a very low dose (25 mg/day).

### Currently ongoing clinical trials investigating CBD for the treatment of ADs (study details)

#### NCT04482244: RCT of CBD for anxiety in advanced breast cancer; active, not recruiting

This double-blind, randomized phase 2 study with parallel assignment uses a single dose of placebo or pharmaceutical grade CBD (Epidiolex^®^), which provides the advantage, as noted previously, of ensuring equal dosing of CBD in each participant. The use of the Anxiety Score-Visual Analog Mood Scale to evaluate anxiety at baseline and post-dosing is a strong point, as most of the available and published data involving CBD and ADs use this scale (Bergamaschi et al. 2011; Linares et al. 2019). Another strong point of the study is that, following dosing, participants will undergo an assessment of tumor burden by computed tomography or positron emission tomography to induce anticipatory anxiety. The secondary outcomes related to adverse effects and measures of pain scores are appropriate for the study population, namely women diagnosed with stage IV breast cancer. The inclusion and exclusion criteria are well thought out and clearly communicated. Although the study is limited to 50 participants, this is considered adequate for this patient population.

However, participants in this study are not required to have a clinical diagnosis of any type of AD. The clinical outcomes measure subjective anxiety associated with personal expectations related to the primary disease of stage IV breast cancer. This study only examined the acute effects of a single dose of CBD, which is appropriate for the research question but has less application to patients with ADs.

#### NCT05600114: Cannabidiol (CBD) for the treatment of social anxiety disorder; active, not recruiting

This active multicenter, double-blind, parallel group, placebo-controlled, randomized phase 2 trial involves 225 participants and is focused on SAD as the primary outcome. The dosing is 300 or 600 mg/day CBD solution for a period of 10 weeks. Inclusion criteria require a clinically predominant diagnosis (DSM-5) of SAD with a Liebowitz Social Anxiety Scale (LSAS) score of 70 or above. The primary outcome is the mean change in LSAS. Neither the secondary outcome measures nor the identity of the CBD solution are disclosed in the NCT. Based on the parameters given in this registration, the study design and disease focus, group size (75 per arm), CBD formulation and dosing, and the study duration appear to be sufficiently powered for a solid result on the primary outcome. Secondary endpoints can only be viewed and discussed once the results have been posted and/or published.

#### NCT05823753: Cannabidiol to reduce anxiety reactivity; recruiting

This short-term study is designed as a randomize, placebo-controlled 3-arm, sub-acute (4-day) steady state dosing phase 2 clinical trial involving 60 participants with clinical diagnosis (DSM-5) of SAD. Eligibility will also require a LSAS score at or above 60 and positive anxiety rating in a public speaking fear questionnaire. The study drug is a pharmaceutical oral solution of CBD (Epidiolex^®^) to be dosed at 300 or 900 mg/day. Outcome measurements focus on the endocannabinoid (anandamide) level and other hematological and urinary endpoints, as well as on state mood assessments, computerized tasks assessing responses to emotional faces, and reactivity to a standardized stress task. Additional questionnaires on stress and anxiety, a driving test, and averse events will be assessed as well. The study aims at understanding CBD effects on the blood levels of anandamide (a stress-regulating endocannabinoid) and the associated anxiety response in the standardized stress task tests. Study completion is intended in April, 2025. Although the group size is relatively small, the stringent eligibility criteria and the standardized testing schedule plus physiological measurements may increase the mechanistic understanding of CBD’s targets and its action on SAD.

#### NCT05753007: A clinical trial of a hemp-derived, high cannabidiol product for anxiety in glioblastoma patients; recruiting

This double-blind, placebo-controlled, 8-week randomized phase 2 trial focuses on anxiety, pain, and quality of life (QOL) in glioblastoma patients who are to undergo treatment with radiation and temozolomide and will use a CBD-rich full-spectrum cannabis extract as adjuvant therapy. The CBD dosage is not indicated, and physiological measurements are not planned. The primary endpoint, anxiety, is assessed using the Overall Anxiety Severity and Impairment Scale and Beck Anxiety Inventory (BAI) tools, both being self-reported questionnaires which may be a limitation in patients with severe cerebral symptoms. Secondary outcomes are pain and QOL to be assessed with seven different questionnaires. Adverse CBD effects and any potential effects on tumor size have not been included as outcome measurements.

#### NCT06364254: Effects of CBD on 2 mile run time trial performance; recruiting

In this randomized, cross-over, placebo-controlled pilot study, the effect of a single dose of CBD (300 mg oral capsule) on pre-race anxiety and difference in gastrointestinal distress (after the run), heart rate variability (after the run), blood lactate levels (during and after the run), and the time to run 2 miles on an indoor treadmill will be measured. As participants, a total of 30 moderately sports-active, trained female subjects are envisaged. The anxiety measurement is based on the 40-items State Trait Anxiety Inventory that shall be completed within 5 min. The scheduled trial completion date is end of May, 2024. According to the study hypothesis is that CBD reduces pre-race anxiety and gut distress which are negatively impacting the exercise performance. In this respect it remains unclear, why gut distress is not also assessed before the run, together with the anxiety questionnaire, but only after the run. Given the known variability in orally administered CBD absorption and hence its bioavailability after a single dosing without control for the mealtime status (hours after intake, fatty or lean food, fasted), one might argue that a four-day treatment to reach a steady state in CBD blood level (as in NCT05823753 described above) would increase the probability for a statistically significant CBD effect.

#### NCT03944447: Outcomes Mandate National Integration with Cannabis as medicine (OMNI-Can); recruiting

This multistate, multicenter, open-label clinical phase 2 study aims at recruiting 200,000 participants from a variety of 33 described conditions, including anxiety. To reach this ambitious number of subjects, the initiators of the study plan to utilize an anonymous online recruitment procedure to evaluate condition(s) for medical cannabis use, cannabis ingestion method, frequency of use, prescription drug use, and demographic information, plus various secondary information. Based on this information, individual CBD treatment recommendations that comply with the applicable state regulations will be given to the patients. If successful, this recruitment would result in a mean number of approximately 6000 participants per condition. The only treatment condition foreseen is medical cannabis preferably administered via RYAH-medtech inhaler in collaboration with OMNI Medical Services, LLC. Medical cannabis strains actually used, product use pattern and patient response will be tracked via phone app. The primary study outcomes as posted on the clinicaltrials.gov website are only specified for COVID-19 prevention and treatment over five years, and for chronic pain over five years. Instruments for the other debilitating diseases, including anxiety, have not been defined. It will be interesting to see, whether this unorthodox, shotgun-like approach is feasible and whether the high number of participants per condition can compensate for the lack of placebo and other controls of patient-specific variables.

#### NCT05457465: Assessing the impact of cannabidiol for anxiety and depression in bipolar disorder; recruiting

This single group assignment, open-label Phase 2 study will investigate the effect of a THC-free, hemp-derived high CBD formulation (to be administered sublingually) on anxiety in patients with bipolar disorder (DSM-5, type I or II) over a 4-week treatment period. A total number of 25 patients is planned to be enrolled. The primary outcome (anxiety) will be assessed using the BAI, and secondary outcome using the Beck Depression Inventory. Other outcome measurements have not been defined for this pilot study which is planned to be completed in June, 2025.

#### NCT05571592: Cannabidiol as a treatment for social anxiety disorder (R61); recruiting

This 3-week randomized, double-blinded placebo-controlled phase 2 trial is planning to enroll 60 participants in two dose groups and one placebo group, respectively. The study drug is an oral capsule containing 400 mg CBD dissolved in a self-emulsifying nanoformulation with enhanced bioavailability (Ananda Scientific), and the dosing will be 400 and 800 mg/day. Inclusion criteria require a DSM-5 diagnosisof SAD and a LSAS score of at least 60. Outcome measures are a public speech test followed by a Visual Analogue Mood Scale at the end of week 2, a standardized 2-day fear learning and extinction protocol at week 3, with functional MRI (fMRI) brain activation accompanying fear extinction recall and fearful faces tasks on the second day, and eleven additional questionnaire-based tools. In addition, plasma levels of CBD will be repeatedly determined immediately before and 1 h after dosing. The planned completion date is August, 2024.

#### NCT04978428: Epidiolex in obsessive compulsive disorder and related disorders; recruiting

This open-label, single-group intervention trial is designed as a proof-of-concept study to test the efficacy and safety of Epidiolex^®^ (pharmaceutical grade CBD). The use of pharmaceutical grade CBD is a strong point of the study. Additionally, the primary and secondary outcomes are well designed, using clinically relevant scales (including the Yale Brown Obsessive Compulsive Scale, National Institute of Mental Health Symptom Severity Scale, the Hoarding Rating Scale, and the Yale Global Tic Severity Scale) to measure efficacy. The inclusion and exclusion criteria are also appropriate for this type of study.

The authors note that this study is outside of the scope of trials involving the effects of CBD on anxiety, as the DSM-5 and ICD-11 no longer consider OCD to be an AD. The design of this study has some limitations, including the absence of a placebo control group and the small number of participants to evaluate the effects of CBD on five different disorders (OCD, trichotillomania, skin picking, Tourette’s disorder, and hoarding disorders). However, given the paucity of data evaluating CBD for the treatment of OCD and OCD-related disorders, this study is quite relevant despite the limitations.

#### NCT03948074: Cannabis for cancer-related symptoms; recruiting

This randomized, double-blind, placebo-controlled study is planned to use a crossover assignment intervention model to assess the efficacy of three different cannabis oils for the management of cancer-related symptoms, including pain, nausea, anxiety, and sleep disturbance. The study is very well designed, given that the literature suggests that formulations containing THC and CBD can help cancer patients during chemotherapy and radiotherapy.

Given the focus of the present review, the main limitation of this study is that subjective anxiety is evaluated as a symptom (assessed by the Patient Global Impression of Change scale for cancer-related symptoms) rather than a disorder. In addition, the study population comprises cancer patients rather than patients with ADs.

#### NCT03549819: Cannabidiol for the treatment of anxiety disorders: an 8-week pilot study; recruiting

NCT03549819 is a randomized, triple-blind, phase 3 trial that uses a parallel assignment model, implying a two-arm trial (CBD or placebo). The trial will be conducted over 8 weeks, which is expected to be an adequate period to evaluate the effects of continuous treatment and possible adverse effects. Participants will be treated with placebo or CBD, starting with one capsule (200 mg) and titrated as tolerated to a maximum of two capsules twice daily (up to 800 mg total dose). The primary outcome measure is the change in Hamilton Anxiety Rating Scale (HAM-A) from baseline to week 8. The HAM-A is a 14-item clinician-based questionnaire to assess general anxiety symptoms (Thompson 2015). Secondary outcomes include changes from baseline to week 8 using a range of behavioral and cognitive scales to evaluate the effects of CBD on general symptoms (e.g., Clinical Global Impression of Severity [CGI-S] and Improvement [CGI-I]); specific ADs, including GAD (assessed by the GAD-7), SAD (assessed by the Liebowitz Social Anxiety Scale-Self Report), panic disorder or agoraphobia (assessed by the Panic and Agoraphobia Scale); and specific symptoms associated with quality of life (e.g., insomnia, assessed by the Insomnia Severity Index), and socio-economic data.

The trial intends to evaluate the effectiveness of a pure CBD-based formulation (brand/source of CBD not disclosed) in four types of ADs: GAD, SAD, panic disorder, and agoraphobia. However, given the placebo-controlled design, a participant size of 50 may limit the ability to achieve statistical power to observe differences between CBD and placebo treatment, particularly if responses vary among the types of AD.

The inclusion/exclusion criteria are appropriate overall; however, patients with MDD are not excluded from the study unless the symptoms are severe. The inclusion of both male and female patients and those up to age 65 may be less advantageous given that ADs occur more frequently in female patients and younger patients.

#### NCT02548559: Sublingual CBD for anxiety disorders; recruiting

This is a randomized, double-blind, placebo-controlled phase 1/2 study that will compare a product containing full-spectrum CBD, a product containing single-compound CBD, and placebo. The primary outcomes of change from baseline at weeks 1, 2, 3, and 4 in the Overall Anxiety Severity and Impairment Scale and self-reported anxiety assessed by the State-Trait Anxiety Inventory are sufficiently complex to detect the effects of CBD in different types of AD and other psychiatric conditions. Primary outcomes are also assessed via the Beck Anxiety Inventory and the HAM-A. The exclusion criteria are appropriate as the study excludes those with serious medical conditions and other neurological and psychiatric disorders.

As a potential limitation, the dose of CBD used in this study (30 mg/day) is low compared with other published studies (Crippa et al. 2018), although we expect that one reason for the relatively lower dose may be to allow for the evaluation of full-spectrum CBD versus single-compound CBD. The study does not single out a specific AD and the number of participants (~ 32) per group is low, which may affect the strength of statistical comparisons. The description of the inclusion criteria is minimal.

#### NCT04075435: Cannabidiol solution for the treatment of behavioral symptoms in older adults with Alzheimer’s disorder; recruiting

This single-group assignment open-label study will evaluate a high CBD/low THC solution (sublingual). Given that there are multiple behavioral symptoms of Alzheimer’s disease (e.g., psychosis, anxiety, seizures, and mood symptoms) and that it is challenging to treat (e.g., patients may require multiple prescriptions, treatments may be poorly tolerated, and BZDs and antidepressants are not recommended), this study has the potential to provide useful and timely data. The primary outcome of this study is to assess the anxiety domain on the Neuropsychiatric Inventory-Clinician scale. Secondary outcomes were evaluated using GAD-7, the agitation and aggression domains of the Neuropsychiatric Inventory-Clinician scale, and the total score of Cohen-Mansfield Inventory.

This study is limited by the open-label design and the small sample size (only 12 patients with Alzheimer’s disease are planned for inclusion), making interpretation of the results somewhat difficult and potentially limiting the impact of the findings.

#### NCT05324449: Epidiolex^®^ for anxiety in pediatric epilepsy; recruiting

This prospective, open-label, single-group study aims to evaluate a standardized pharmaceutical grade formulation of CBD (99.99% CBD, 100 mg/mL; GW Pharmaceuticals, Cambridge, UK), this ensures that all participants receive the same dose of CBD. This is the only trial identified in our search that investigates CBD in children and teenagers aged 6–17 years. The inclusion and exclusion criteria are well described for this study.

This trial evaluates the treatment of anxiety secondary to refractory pediatric epilepsy, meaning that ADs are not the primary focus of the study. The primary endpoint being related to changes in CGI-I from baseline is not ideal because this scale was designed for clinicians only, to capture the screening clinician’s impression about the severity or improvement of a patient’s symptoms (Busner & Targum 2007). Given the open-label, single-arm design of the study, it may be difficult to evaluate the efficacy of CBD as an adjunctive therapy to treat anxiety in pediatric patients with epilepsy. Screening anxiety treatment in only 30 pediatric patients is another possible weakness of this trial.

#### NCT02283281: Anesthetic premedication with cannabis extracts (Cannapremed); recruiting

This randomized, placebo-controlled (parallel assignment with three arms) phase 2/3 trial plans to enroll an adequate number of participants (*N* = 200) to evaluate the efficacy of a CBD:THC formulation (1:1; similar to Sativex^®^). The pre-operative doses (21.6 mg THC + 20 mg CBD or 10.8 mg THC + 10 mg CBD) are administered intravenously as a single bolus.

In the context of evaluating CBD for the treatment of ADs, we note that the primary outcome of the study is the effect of CBD on post-operative pain. Anxiety is a secondary outcome monitored as a post-operative symptom (self-assessed, using a visual analogue scale). Thus, the study was not designed to evaluate the effects of CBD or other cannabinoids on ADs.

#### NCT04878627: Role of CBD in regulating meal-time anxiety in anorexia nervosa; recruiting

This double-blind, placebo-controlled (parallel assignment) phase 1 study is set to include 40 participants, which is expected to appropriately support evaluation of the study outcomes. In contrast to most of the studies in this review, the study design is of high quality. The primary outcomes, which include evaluation of adverse effects, testing for CBD metabolites in the blood, and change from baseline in the Eating Disorder Examination Questionnaire scores over the course of treatment (weekly over 3 weeks) are also considered appropriate. The study design and inclusion/exclusion criteria are pertinent and well described. The follow-up is considered adequate. A final strong point of this study compared with some of the other studies discussed herein is that participants will receive pharmaceutical grade CBD (oral).

Participants will receive escalating doses of CBD over the 3-week study; none of the participants will receive a fixed dose for the duration of the study, which may represent a limitation of this study.

#### NCT05283382: Cannabidiol effects on learning and anxiety; not yet recruiting

This is a randomized, double-blind, placebo-controlled trial that will use two-by-two factorial assignment. The number of participants is planned to be 160 and participants will be treated with a one-time dose of placebo or CBD (600 mg) in the form of six gel capsules (brand/source of CBD not disclosed).

The trial accepts healthy volunteers who display elevated social anxiety. Given that participants are given a one-time treatment, only the acute effects of CBD can be evaluated. The main focus of the study is to determine whether CBD facilitates enhanced extinction compared with placebo. The primary outcome of electrodermal response is poorly connected to ADs and highly connected to subjective anxiety (Birket-Smith et al. 1993; Jensen et al. 1996). No secondary measures are planned. The application of a self-reported fear visual analogue scale is mentioned but not categorized as a secondary measure. The inclusion and exclusion criteria for this study are minimal and somewhat unclear as the study description mentions that the effects of CBD will be evaluated in participants who show elevated social anxiety, but this is not mentioned in the inclusion/exclusion criteria.

#### NCT06290063: Cannabidiol and older adult cannabis users (QUARTz); not yet recruiting

This study is designed as a triple-blinded, randomized, 3-arm placebo-controlled Phase 2 trial with 285 participants. The effect of CBD on sleep, anxiety, depression, and pain will be investigated in an elderly target population (aged 60 years or above). The eight-week treatment will consist of hemp-derived full-spectrum extract (200 mg/day CBD, 4 mg/day THC), hemp-derived broad spectrum extract (200 mg/day CBD, no THC), or placebo. Eligibility criteria require that the patients are currently taking medication/s for pain, sleep, and/or mood; however, a clinical diagnosis of the condition is not requested. Multiple primary outcome measures are foreseen, including three instruments for anxiety and depression: the Beck Depressin inventory, Beck Anxiety Inventory, and Depression Anxiety Stress Scale. The inflammatory state will be assessed by measuring blood levels of Interleukin-6, TNFα, Interleukin-1β, and c-reactive protein (CRP). Study completion is planned for July, 2028. Although the group size of approximately 130 individuals per arm can provide substantial statistical power, the somewhat diffuse criteria for the participants’ health conditions may reduce the stringency of the expected outcomes.

#### NCT06266611: Cannabis for Palliative Care in Cancer (ARCTiC); not yet recruiting

This study has been designed by the same group as the previously described study, NCT06290063. Accordingly, it is planned as a triple-blinded, randomized, 3-arm placebo-controlled Phase 2 trial, and uses the same CBD formulations and doses: hemp-derived full-spectrum extract (200 mg/day CBD, 4 mg/day THC), hemp-derived broad spectrum extract (200 mg/day CBD, no THC) over an eight-week treatment period. The effect of CBD on sleep, pain, mood (depression, anxiety), cognitive functions, and QOL will be compared with placebo in cancer patients having symptoms of the above listed disorders, and the target number of participants per group is approximately 62 per arm, 185 patients in total. Eligibility criteria include previous or current curative or palliative solid tumor treatment during the past 18 months and current symptoms of sleep disturbance, pain, anxiety, and/or depression. More than 20 primary outcome measures will be applied, but only the Depression Anxiety Stress Scale – 21 will address anxiety and depression symptoms. As discussed for the sister study, the lack of focus in the CBD target symptoms may limit the power of this trial, in spite of its above-average number of patients. Estimated study completion date is also July, 2028.

#### NCT06123702: Cannabidiol effects on fear extinction in social phobia; not yet recruiting

This double-blind, randomized controlled Phase 1 trial will assess the effect of CBD vs. placebo for potentiating fear extinction in adult outpatients with SAD. 20 SAD patients (DIAMOND grade 4 or higher) will be recruited and will receive a single dose of 600 mg CBD or placebo after the fear conditioning trial. Conditioning will achieved by combining pictures of angry faces with an unpleasant electrical stimulus to the wrist. During the following fear extinction phase, the angry faces are shown without electric stimulus. The degree of fear will be measured objectively leveraging the galvanic skin reaction (which may be poorly connected to ADs and highly connected to subjective anxiety, as laid out above, and subjectively using a visual analog scale for the discomfort/fear when seeing the conditioned angry faces during the extinction phase. The study hypothesis is that CBD decreases galvanic skin reaction and subjective fear ratings. This basic research study addresses a mechanism of fear extinction which may not be specific to SAD patients but can also be relevant to other forms of anxiety and to post-traumatic stress disorder.

#### NCT05649059: Investigating the effects of Cannabidiol on Social Anxiety Disorder (CAN-SAD); not yet recruiting

This is another mechanistic acute exposure study leveraging an experimental model of SAD, designed as a randomized, double-blind, placebo-controlled, parallel-group phase 4 trial. The study drug will be pure CBD oral solution (Epidiolex^®^, 300 mg/kg), administered as a single dose. 50 patients with clinically confirmed SAD (QuickSCID-5, LSAS ≥ 60) will be randomly allocated to CBD or placebo. Three hours after drug administration, the Trier Social Stress Test (TSST) will be conducted. According to the study description, the TSST is the gold-standard for ethically inducing stress in a controlled laboratory setting. Physiological stress and emotional processing will be measured by brain fMRI and salivary alpha-amylase levels at various time-points before and after the TSST, and stress-induced anxiety will be subjectively assessed using a modified Visual Analog Mood Scale. The anticipated study completion date is May, 2025.

#### NCT06261502: Effect of CANnabidiol on Anxiety and GABAergic function in individuals with fragile-X syndrome (CANAX); not yet recruiting

This randomized, double-blind, placebo-controlled, single center, cross-over Phase 2 trial will assess the effect of oral CBD on symptoms of autism spectrum disorder (ASD) and possibly causal γ-aminobutyric acid (GABA) signaling in the brain in individuals with the fragile X syndrome. An estimated 40 participants will be enrolled for this 2 × 12 weeks plus 8-week washout crossover study. Eligibility criteria include a molecular diagnosis of fragile X and an overall ABC-C score > 20. Patients will receive an oral CBD solution (possibly Epidiolex^®^, since Jazz Pharmaceuticals is a study collaborator) with a dose escalation from 5 mg/kg/day to 10 mg/kg/day). Primary outcome measures will be the Anxiety, Depression, and Mood Scale (ADAMS) to be completed by the caregivers of the patients, and two more tools to assess disruptive behavior. Secondary outcomes are noninvasive, NMR-based GABA concentration measurements in the brain and transcranial magnetic stimulation-derived measure of intracortical inhibition and facilitation. The results are expected to corroborate that CBD can improve the GABAergic function impairment underlying anxiety in this specific patient population, however such mode of action may also be relevant to the large spectrum of mood disorders in which GABAegic inhibition plays a causal role, e.g., other forms of anxiety, major depression, postpartum depression, and premenstrual dysphoric disorder (Thompson 2024). The planned study completion date is August, 2027.

### Abandoned clinical trials investigating CBD for the treatment of ADs

#### NCT05429788: Safety, tolerability, and efficacy of RLS103 in subjects with acute anxiety with social anxiety disorder; withdrawn

This randomized, double-blind, placebo-controlled (parallel assignment) phase 1b/2a trial aims to determine the effects of a CBD inhaled powder (Technosphere^®^), using a very specific model of acute anxiety induced by simulated public speaking, in participants with SAD. A single dose of CBD (3 mg or 6 mg) was administered prior to a public speaking challenge. The choice of both the primary outcome of incidence and severity of adverse events and the secondary outcomes of change in participant-reported anxiety (Subjective Units of Distress Scale) and Clinical Global Impression of anxiety improvement are supported by previously published findings (Bergamaschi et al. 2011; Linares et al. 2019). The innovative delivery method via inhalation emulates that used to deliver corticosteroids for asthma and respiratory allergies. The study was designed to evaluate participants acutely, but also includes a follow-up of 4–6 weeks. We note that this study has some similarities with NCT04086342, which was mentioned previously, but has been withdrawn because of pipeline changes.

The basis for the CBD dose levels chosen for this study (3 mg and 6 mg) is unclear; the doses may be considered somewhat low to achieve a measurable effect. It would be of interest to include groups receiving the same CBD doses via the oral route to compare the effectiveness of the two routes of administration in acute anxiety in patients with SAD. Moreover, the effective doses (i.e., plasma levels of CBD/metabolites) should be determined. It would also be beneficial to have a larger study population of 60 participants rather than the planned 30 participants, given that there are three treatment arms.

#### NCT04267679: Cannabidiol for anxiety; withdrawn because of the COVID-19 pandemic

This primary outcome in this study was planned to be assessed using the GAD-7, which is the most frequently used diagnostic assessment for GAD. The study duration was 12 weeks, which would be expected to be sufficient to evaluate the effects of repeated dosing.

This was planned to be a single-group assignment, open-label phase 2 trial using a low dose of full-spectrum CBD (25 mg). The number of planned participants and exclusion criteria are not clear from the listing on ClinicalTrials.gov.

#### NCT04286594: A clinical trial of hemp-derived CBD product for anxiety; terminated by sponsor

The main strong point of this study is its randomized, double-blind, placebo-controlled (parallel assignment) design.

A potential limitation is that this study used whole plant extract containing 30 mg/mL CBD rather than a pharmaceutical grade formulation. Additionally, the daily dose of CBD (sublingual) is 25 mg/day, which is considered a low dose (Crippa et al. 2018).. The primary outcome is based on changes in a self-reported anxiety measure by the Beck Anxiety Inventory, which may facilitate the placebo effects well reported in human studies involving psychiatric conditions and cannabinoids as treatments (Gedin et al. 2022; Hodgins et al. 2018).The inclusion criteria indicate that clinical assessment and a specific type of AD were not required for study participation.

#### NCT04726475: Impact of CBD-rich oil on aversive memory reconsolidation; unknown status

This three-arm, proof-of-concept double-blind, placebo-controlled trial using CBD-rich oil is well designed and based on preclinical data from the literature. Given the study title, it is assumed that this study aims to investigate possible uses of CBD in patients with PTSD.

The primary outcome measure is CO_2_ emotional reactivity, with secondary outcomes intended to be assessed by CO_2_ emotional distress recovery trajectory and the Short Scale Anxiety Sensitivity Index. The 35% CO_2_ interoceptive memory reactivation procedure is known as a potent inducer of panic attacks. It is unclear from the inclusion criteria listed whether healthy participants or participants with PTSD or ADs are the target study population. It is also not clear why the intervention of 300 mg CBD-rich oil is considered a dietary supplement; however, this may indicate that the CBD used will not be pharmaceutical grade.

#### NCT04729244: The study of hemp oil CBD for evaluation of efficacy and safety in treatment of pain, anxiety, and insomnia management; unknown status

This non-randomized, open-label pilot study of patients with chronic pain currently using analgesics (healthy volunteers also accepted) plans to compare two formulations of CBD (dermatological cream or orally administered CBD oil). The use of the HAM-A scale to evaluate change in anxiety from baseline to end of study (4 weeks) is a strength of the study. Additionally, the number of participants included and the inclusion/exclusion criteria appear to be sufficient and appropriate to support the study aim.

However, the non-randomized, open-label design and inclusion of healthy volunteers may pose some limitations. The dose of CBD is planned as 50 mg, which is appropriate for the management of pain but considered to be a low dose for the treatment of ADs (Crippa et al. 2018). Furthermore, anxiety is considered a symptom in this study rather than the primary disorder, as this is a study of the effects of CBD in patients with chronic pain.

#### NCT04569760: Cannabidiol for the treatment of anxiety disorders: an 8-week pilot study; withdrawn (study cancelled prior recruiting)

This trial was designed as a randomized, double-blind, placebo-controlled (parallel assignment), randomized phase 3 study to determine the effectiveness of a formulation of 50:2 mg/mL (CBD:THC) administered orally to treat GAD, SAD, panic disorder, and agoraphobia. The primary and secondary outcomes were well defined (including assessments via GAD-7, HAM-A, CGI-S, and CGI-I) as were the inclusion/exclusion criteria. The planned 8-week study duration was appropriate, and it was planned that participants would be evaluated by physicians at least six times during the study.

It was planned that participants could receive up to 800 mg CBD and 8 mg THC daily if tolerated, which is considered a high dose for both compounds. Considering that young adults (21 years old) were planned to be included in the study, such a high daily dose of THC may be problematic (Crane & Phan 2021). The high (800 mg) dose of CBD may not yield detectable effects if the reported bell-shaped dose–response curve would apply to this study population and study design. The study planned to enroll 25 participants per group. This participant number may have been inadequate to evaluate changes in participants with four different ADs. Participants were neither required to be on pharmacological treatment for their AD nor required to not be receiving pharmacological treatment, which may have made interpretation of the results somewhat difficult.

#### NCT04086342: CHI-902 for treatment of social anxiety disorder; withdrawn due to pipeline changes

This double-blind, placebo-controlled (parallel assignment) phase 2b study was well designed and planned to evaluate a standardized enriched CBD extract (CHI-902) in patients with SAD. The planned doses (150 mg to 300 mg/day, escalated over time) were in line with previous reports (Crippa et al. 2018). The investigators planned to include 160 participants. The primary endpoint of change in the Liebowitz Social Anxiety Scale from baseline to 8 weeks and secondary endpoints of monitoring adverse effects are appropriate to support the aim of the study. Endocannabinoid levels were planned to be assessed during the study. The inclusion and exclusion criteria met gold standards for this type of clinical trial.

Given that this was a dose-escalation protocol, there were no participant groups that were planned to receive a stable dose over the 3-week study. We note that SAD is more prevalent in teenagers and young adults; therefore, the age allowance of participants may be considered too wide (≥ 18 years of age).

## Conclusions and perspectives

The majority of the recently completed and currently ongoing clinical trials are exploratory and do not focus on specific types of clinical anxiety or AD as the primary condition studied. The studies are very heterogeneous in several key aspects, including CBD dose, CBD formulation, treatment duration, and number of participants. Many trials have a low number of participants and only two of them are multicenter trials, which would be of benefit.For comparison, regulatory agencies based their approval of Epidiolex^®^ to treat Dravet syndrome (DS) and Lennox-Gastaut syndrome (LGS) on four essential double-blind, placebo-controlled, RCTs, involving a total of 154 DS patients and 396 LGS patients. In these trials, reduction in the monthly frequency of seizures as the primary outcome measure was achieved with two doses of CBD, 10 or 20 mg/kg/day BID (Morano et al. 2020). Concerning the potential efficacy of CBD to treat AD, only two of the six completed trials have reported results. They do not support an anti-anxiety effect of CBD, while the primary endpoint was on motor symptoms in Parkinson’s disease patients. Also in the remaining four completed trials with not yet published results, their design parameters and experimental conditions are not indicative of a strong evidence for or against CBD as a medication to treat AD. Although currently a total number of 22 clinical trials on CBD in AD is ongoing, our evaluation of their design and experimental parameters, compared with those from the completed and abandoned trials, is not very likely to accomplish the necessary body of solid clinical evidence that is required to move the therapeutic use of CBD in AD patients forward.

Considering the current state of the art in AD treatment, the authors propose that the development of appropriately designed clinical trials in the following indications to evaluate the clinical effects of CBD would be indispensible:

- SAD: There is a good level of experimental evidence in the literature related to the treatment of SAD with CBD (Bergamaschi et al. 2011; Berger et al. 2020; Linares et al. 2019; Wright et al. 2020). Given that the currently approved pharmacological treatment with selective serotonin reuptake inhibitors (paroxetine, sertraline, and fluvoxamine) may leave an unmet need for some of these patients, and that the prevalence has increased as a result of the COVID-19 pandemic, such trials appear quite timely. Out of the ongoing trials, seven are focusing on SAD, four of them in acute settings (single dose or 4 days), and the other three over four to ten weeks treatment; the doses are in the mid-to-high range (150 to 900 mg/day). A four-day dosing schedule before the acute anti-SAD assessment may reduce variability in the endogenous CBD concentration (plasma levels to be confirmed), and a highly standardized stress/anxiety test protocol such as the carbon dioxide challenge or Trier test may result in a reliable primary outcome measure. Ideally, the questionnaire-based SAD assessment tools will be combined with objective measurements including physiological parameters such as cerebral fMRI or NMR-based GABA concentration measurements. SAD could also be studied as a secondary condition in children who are receiving CBD to reduce seizures or to treat autism spectrum disorders.
- GAD: Two registered and currently ongoing clinical trials evaluating CBD in ADs are focused on GAD, and six on AD forms associated with neurodegenerative diseases, anorexia, and cancer. GAD could be further investigated as a third arm in planned trials for patients with SAD.
- PTSD and related disorders: Evidence for the effectiveness of CBD in patients with PTSD and related disorders is low; therefore, clinical trials of CBD in this indication would be considered high risk and would need to be initiated as exploratory studies and followed up, if warranted, by a double-blind, open-label, phase 1/2 mixed model study. The authors note that in such studies, the nature of the trauma should be considered relevant. For example, in the case of an exploratory clinical trial, a mixed model of contextual fear-induced stress based on a virtual reality model could be appropriate. In phase 2 studies, the most appropriate primary outcome would be changes in the Clinician-Administered PTSD Scale for DSM-5.
- Panic disorder: As with PTSD, the evidence for the efficacy of CBD treatment in patients with panic disorder is limited. Therefore, the approach to clinical trials would be the same. Some fear conditioning and extinction paradigms as applied in a few SAD trials may also address mechanisms relevant to PTSD patients. Of note, it is important to differentiate between panic disorder with or without agoraphobia.
- OCD, specific phobias, and other ADs: There is little available evidence of the efficacy of CBD in patients with OCD, specific phobias, or other ADs. Exploratory studies that include few patients to evaluate CBD as an add-on therapy may be most appropriate.

While currently (April 2024) 22 studies on the effect of CBD on anxiety are both planned and underway, there are only two completed studies (none of them designed as a double-blind RCT) with a sufficient number of participants to evaluate the effect of CBD on specific ADs. The results of the currently planned/ongoing studies are eagerly anticipated, as are new well-designed studies to evaluate the effects of standardized CBD formulations on specific ADs, as well as systematic investigations into CBD formulations and delivery methods.

## Data Availability

Not applicable.

## Abbreviations

AD: Anxiety disorder
ADAMS: Anxiety, Depression, and Mood Scale
BAI: Beck Anxiety Inventory
BID: *bis**in die* (Two times daily)
BZD: Benzodiazepine
CBD: Cannabidiol
CGI-S: Clinical Global Impression of Severity
CRP: C-reactive protein
DALY: Disability-adjusted life year
DS: Dravet syndrome
DSM-5: Diagnostic and Statistical Manual of Mental Disorders, 5th edition
fMRI: Functional magnetic resonance imaging
GAD: Generalized anxiety disorder
GAD-7: Generalized anxiety disorder assessment
HAM-A: Hamilton Anxiety Rating Scale
GABA: γ-Aminobutyric acid
ICD-11: International Classification of Diseases
LGS: Lennox-Gastaut syndrome
LSAS: Liebowitz Social Anxiety Scale
MDD: Major depressive disorder
NICE: National Institute for Health and Care Excellence
OCD: Obsessive–compulsive disorder
PTSD: Post-traumatic stress disorder
QOL: Quality of life
RCT: Randomized controlled trial
SAD: Social anxiety disorder
SNRIs: Selective serotonin-noradrenalin reuptake inhibitors
SSRIs: Selective serotonin reuptake inhibitors
THC: Δ9-Tetrahydrocannabinol
TSST: Trier Social Stress Test
US: United States of America
WHO: World Health Organization
